# Detection of methyl parathion using a novel oxidized polylevodopa fluorescence nanosensor

**DOI:** 10.3389/fchem.2026.1849994

**Published:** 2026-08-06

**Authors:** Delai Zhou, Jian Xu, Fankui Zeng, Fude Yang

**Affiliations:** 1 College of Pharmacy, Gansu University of Chinese Medicine, Lanzhou, China; 2 Research Center for Natural Medicine and Chemical Metrology, Lanzhou Institute of Chemical Physics, Chinese Academy of Sciences, Lanzhou, China

**Keywords:** fluorescent sensor, inner filter effect, methyl parathion, oxidized polylevodopa, pesticide detection

## Abstract

**Introduction:**

An ultra‐sensitive fluorescence sensing system was constructed for methyl parathion (MP) detection using oxidized polymer of levodopa (L‐DOPA) as a novel fluorescent probe.

**Methods:**

L‐DOPA was first polymerized under alkaline conditions to form black L‐DOPA polymer (PDOPA), and then H_2_O_2_ was added to oxidize it to form oxidized polymer of L‐DOPA (OPDOPA), which exhibits strong blue fluorescence. Based on the alkaline‐catalyzed hydrolysis of MP and the inner filter effect (IFE) between its hydrolysis product p‐nitrophenol (p‐NP) and OPDOPA, the detection of MP was successfully achieved.

**Results:**

The OPDOPA fluorescence nanoprobe presents excellent selectivity and sensitivity for MP detection. A linear response was achieved from 0.5 to 30 µg/mL, and the limit of detection was determined to be 0.08 µg/mL. The fluorescence detection system was utilized for the determination of MP in potato and Codonopsis pilosula samples. For the actual analysis of the two sample matrices, the sensor exhibited recovery rates spanning 92.00%–106.42% and a relative standard deviation (RSD) of ≤5.83%.

**Discussion:**

In this study, a functional polymer material with excellent fluorescence properties was synthesized from L‐DOPA and applied to the highly sensitive and selective detection of MP. This approach not only offers a novel strategy for the detection of organophosphorus pesticides but also substantially broadens the application horizon of functional polymer materials.

## 1 Introduction

As a highly effective, broad-spectrum insecticide, MP is commonly prepared as emulsifiable concentrates or powders for pest control in agriculture (Song et al., 2017). Meanwhile, as an organophosphorus pesticide intended only for outdoor use, MP is designated as toxicity category I by the United States Environmental Protection Agency. (Rubin et al., 2002). In modern toxicological research, the toxic effects of MP and its metabolites on invertebrates, vertebrates, and some microorganisms have been confirmed. MP can enter organisms through multiple pathways, which may involve the skin, eyes, mucous membranes, and respiratory tract (Cáceres et al., 2019; Castrejón-Godínez et al., 2022; Chen et al., 2023). Moreover, it elicits toxic effects in humans and animals by inhibiting synaptic acetylcholinesterase, and evidence of certain genotoxicity has been observed (Aroniadou-Anderjaska et al., 2023; Patnaik and Padhy, 2016). Given the extensive application of MP in agricultural production and household pest control, residues of this organophosphorus pesticide are widely present in food, herbal medicines, and the environment (Sang et al., 2024; Wang et al., 2021; Witczak et al., 2018). In summary, it is crucial to establish a rapid and efficient strategy for the detection of MP residues in food, environmental matrices, and herbal medicinal materials.

Currently, various methods, including high-performance liquid chromatography, gas chromatography, gas chromatography-mass spectrometry, surface-enhanced Raman spectroscopy (SERS), and enzyme-linked immunosorbent assay (ELISA), have been applied for the detection of MP. However, their practical applications are restricted due to the inherent drawbacks of these techniques (Zhang et al., 2023). For instance, chromatography and chromatography-mass spectrometry methods suffer from high instrumental costs, complicated and time-consuming sample pretreatment procedures, as well as the requirement for professional operators (Chikte et al., 2024). ELISA is highly dependent on specific antibodies (Ge et al., 2026). For SERS, the stability is easily compromised owing to the complicated preparation process of its substrates (Xie et al., 2020).

As an essential component of real-time sensing technologies, fluorescent probes have gained extensive development in MP detection, benefiting from their inherent advantages of high sensitivity, excellent selectivity, and real-time monitoring capability. Notably, a subset of these probes achieves MP detection by relying on the catalytic action of enzymes (Li R. et al., 2025; Song et al., 2017). However, the reliance of such research on enzymes, coupled with the susceptibility of enzymatic activity to external conditions, has impeded the practical application of this technology. In contrast, alternative studies have focused on the indirect determination of MP by inducing its hydrolysis to produce p-NP. Typical examples include fluorescent probes fabricated based on carbon dots (CDs) and silicon quantum dots (SiQDs), and these studies collectively demonstrate that detecting MP via its hydrolysis products is a feasible and effective strategy (Yang et al., 2025; Zhang et al., 2023).

**SCHEME 1:**
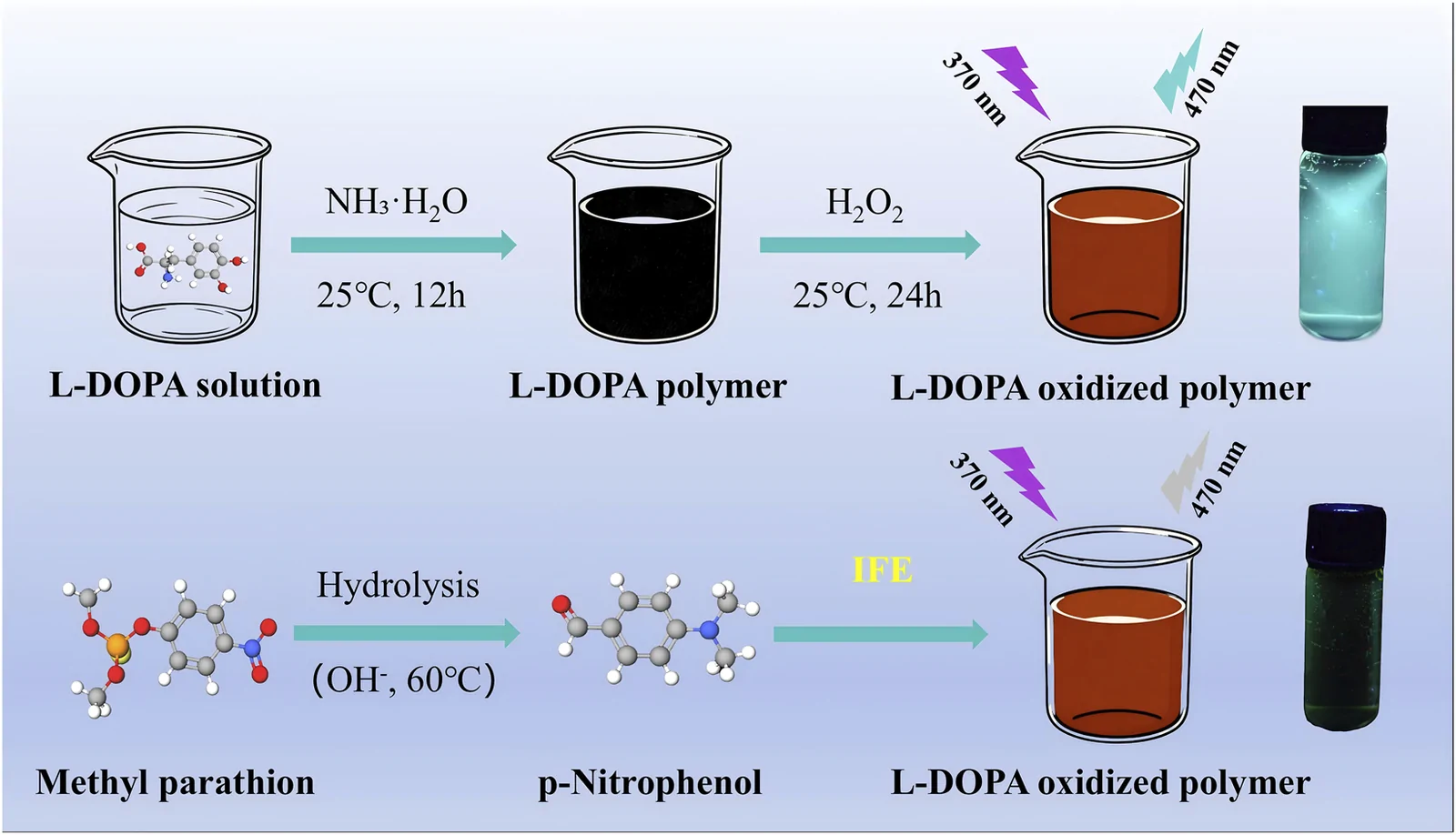
Schematic illustration of the synthesis and fluorescence changes of L‐DOPA polymers. The upper panel shows that L‐DOPA solution reacts with ammonia at 25 °C for 12 h to form a black polymer, which is then reacted with hydrogen peroxide at 25 °C for 24 h to yield an orange oxidized polymer. Upon excitation at 370 nm, it emits blue fluorescence at 470 nm. The lower panel displays the hydrolysis of methyl parathion into p‐nitrophenol, which quenches the blue fluorescence of the polymer via the inner filter effect, resulting in a dark vial. Molecular structures and reaction conditions are depicted.

Here in, a novel fluorescent sensor based on OPDOPA was successfully prepared. Using OPDOPA as the nanosensor, a simple, highly sensitive, and rapid enzyme-free biosensing system was established for the detection of MP in foods and traditional Chinese medicines (Scheme 1). The linear range, sensitivity, and selectivity of the proposed method were evaluated, and its detection mechanism was explored. Finally, the feasibility of this method was verified in actual samples of potato and *Codonopsis pilosula*. In summary, the sensor adopts an enzyme-free design and optimizes hydrolysis conditions, thereby providing a novel solution for the detection of MP in food and traditional Chinese medicines. Compared with conventional detection methods, the strategy exhibits advantages of low cost, high safety, and simple operation.

## 2 Materials and methods

### 2.1 Instruments and equipments

Fluoromax-4 Fluorescence Spectrophotometer (HORIBA, Ltd., Japan), Lambda750 Ultraviolet-Visible Spectrophotometry (UV-Vis, PerkinElmer, Inc., United Kingdom), Bruker V70 Fourier Transform Infrared Spectrometer (FTIR, Bruker Corporation, Germany), ESCALAB 250Xi X-ray Photoelectron Spectrometer (XPS, Thermo Fisher Scientific Inc., United States), JSM-6701F Field Emission Scanning Electron Microscope (SEM, JEOL Ltd., Japan), PHS.2C precision pH meter (Mettler-Toledo International Inc., United States), Centrifuge 5810 R high-speed refrigerated centrifuge (Eppendorf SE, Germany) were used.

### 2.2 Materials and reagents

MP, methamidophos, and chlorpyrifos were obtained from Bailingwei Technology Co., Ltd. (Beijing, China). Dimethoate and dichlorvos were obtained from Alta Scientific Co., Ltd. (Tianjin, China). Glyphosate, fenthion, and phoxim were supplied by Yuanye Bio-Technology Co., Ltd. (Shanghai, China). L-DOPA was acquired from Macklin Biochemical Technology Co., Ltd. (Shanghai, China). Ethanol and hydrogen peroxide were obtained from Kelong Chemical Co., Ltd. (Chengdu, China). Acetonitrile (HPLC grade) was purchased from Merck KGaA (Darmstadt, Germany). Ultrapure water was used in all experiments, and all chemicals were of analytical grade or higher and used as received without further purification.

### 2.3 Synthesis of OPDOPA nano-fluorescent probe

OPDOPA was synthesized according to the method reported by Ma et al., with appropriate modifications (Ma et al., 2018), and the detailed procedure is described below.

At room temperature, 0.25 g of L-DOPA was dispersed in 70 mL of ethanol-water mixture (volume ratio = 2:5). After magnetic stirring for 10 min to achieve sufficient dispersion, 1 mL of NH_4_OH was added dropwise to the system, followed by constant stirring for 12 h to afford PDOPA. Subsequently, under continuous magnetic stirring, 18 mL of H_2_O_2_ was slowly and uniformly introduced into the mixture. The reaction was sustained with stirring for an additional 24 h. After dialysis in the dark for 24 h (1,000 Da), the OPDOPA solution was finally prepared. All reactions described above were performed at room temperature under light-proof conditions.

### 2.4 Detection of MP

MP solutions with various concentrations were hydrolyzed in 100 mM NaOH at 60 °C for 40 min to generate p-NP. Subsequently, the pH of the resulting hydrolysate was tuned to 7.0 with HCl (0.1 M), and PB buffer (100 mM, pH 7.0) was introduced to bring the total volume to 4.0 mL. Finally, 50 μL of OPDOPA solution was added to the system. After vortex mixing, the mixture was incubated at room temperature in the dark for 10 min prior to measurement. Fluorescence emission spectra were recorded at an excitation wavelength of 370 nm. The linear correlation between (F_0_−F)/F_0_ and the concentration of MP was established based on the emission spectra, where F_0_ is the fluorescence intensity of the MP-free system at 470 nm, and F is the fluorescence intensity at 470 nm after the addition of MP.

### 2.5 Real sample analysis

Commercially available potato and *C. pilosula* were selected as experimental samples, and the specific pretreatment procedure was as follows.

Potato samples: Potato samples were thoroughly homogenized at high speed using a homogenizer. Accurately weighed 60 g portions of the homogenate were transferred into a centrifuge tube. Appropriate volumes of an MP standard working solution were added to achieve final spiked concentrations of 0.01, 0.02, and 0.04 mg/kg. After vortex mixing, 60 mL of acetonitrile was added as the extraction solvent, followed by vigorous shaking for 10 min. Subsequently, 27 g of anhydrous magnesium sulfate (MgSO_4_) and 9 g of anhydrous sodium acetate (NaOAc) were added. The mixture was immediately vortexed for 1 min and then centrifuged at 10,000 × g for 10 min at 4 °C. The total supernatant was collected, and its volume was recorded. Sorbent materials were added at a ratio of 10 mg of primary secondary amine (PSA) and 5 mg of octadecylsilane (C_18_) per milliliter of supernatant. After vortex mixing for 2 min, the mixture was centrifuged again under the same conditions (10,000 × g, 4 °C) for 10 min. The resulting supernatant was transferred to nitrogen evaporation tubes and concentrated to near dryness using a gentle stream of nitrogen gas in a 40 °C water bath. After cooling to room temperature, the residue was redissolved in 0.5 mL of acetonitrile by vortex mixing for 1 min. The resulting solution was then subjected to MP quantification as described in Section 2.3. All experiments were performed in triplicate to ensure data reliability.

*Codonopsis Radix* samples: A precisely weighed 60 g sample was placed into a centrifuge tube. A suitable volume of acetone was added to fully moisten the powder, and aliquots of MP standard solution were spiked into the matrix to achieve final MP concentrations of 0.01, 0.02, and 0.04 mg/kg. Subsequently, 60 mL of ultrapure water was added, and the mixture was allowed to stand for 10 min. After that, 120 mL of acetonitrile was introduced, and the mixture was extracted by shaking for 10 min. Then, 27 g of MgSO_4_ and 9 g of NaOAc were added to the extract, followed by vortexing for 1 min to ensure thorough mixing. The mixture was centrifuged at 10,000 × g for 10 min at 4 °C. The total supernatant was collected, and its volume was recorded. Sorbent materials were added at a ratio of 10 mg of PSA and 5 mg of C_18_ per milliliter of supernatant. After vigorous vortexing for 2 min to ensure thorough dispersion, the mixture was centrifuged at 10,000 × g for 10 min. The purified supernatant was then transferred to a nitrogen evaporation tube and concentrated to near dryness under a gentle stream of nitrogen at 40 °C. After cooling to room temperature, the residue was redissolved in 0.5 mL of acetonitrile by vortex mixing for 1 min. The resulting solution was then subjected to MP quantification as described in Section 2.3. All experiments were performed in triplicate to ensure data reliability.

## 3 Results and discussion

### 3.1 Characterization of OPDOPA

We have systematically characterized OPDOPA and its intermediates from various aspects, including morphology, particle size, types of functional groups, surface electronic states, and optical properties.

**FIGURE 1:**
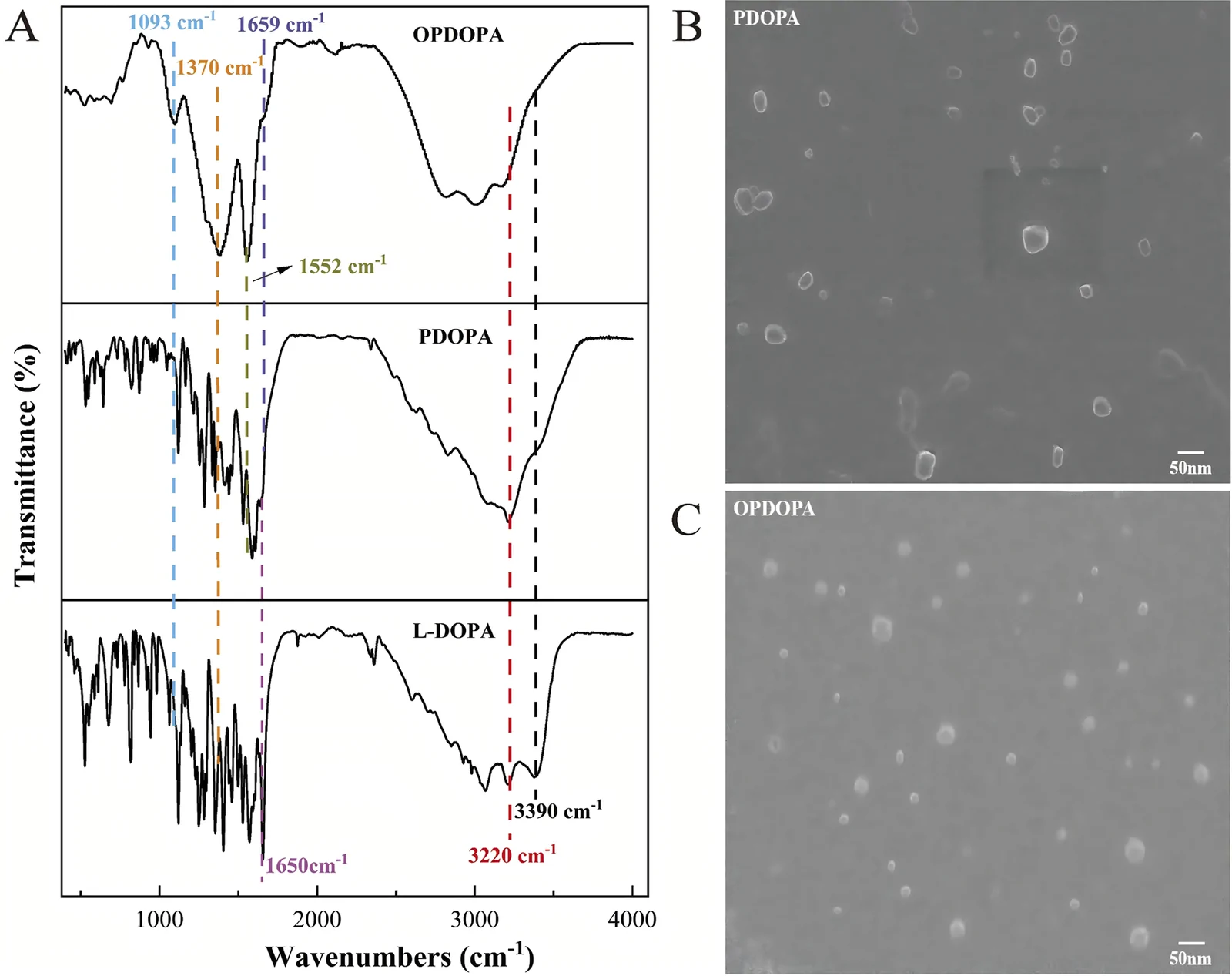
**(A)** FTIR spectra of L-DOPA, PDOPA, and OPDOA. **(B)** SEM images of PDOPA. **(C)** SEM images of OPDOPA.

As depicted in Figures 1B,C, OPDOPA exhibits a more uniform distribution and a more regular morphology compared to PDOPA. Statistical analysis of their particle size distributions (Supplementary Figure S1) revealed that OPDOPA exhibited a size distribution ranging from 7 to 36 nm, with a mean diameter of 18 nm. In contrast, PDOPA particles had a size range of 7–47 nm and an average diameter of 20 nm. The FTIR spectrum of L-DOPA was in good agreement with the results reported in previous studies (Figure 1A) (Edwin and Hubert Joe, 2013). Under alkaline conditions, L-DOPA can undergo polymerization, which induces structural changes (Hemmatpour et al., 2023). In this work, distinct differences were observed between the FTIR spectra of PDOPA and L-DOPA. Specifically, the stretching vibration intensity of the N-H bond (3,390 cm^-1^) and the bending vibration intensity (1,650 cm^-1^) in PDOPA were attenuated, while the stretching vibration intensity of the C-N bond (1,370 cm^-1^) was enhanced. These findings indicate that the NH_2_ group in L-DOPA is converted into C-N bonds during polymerization, which can be ascribed to the cyclization reaction occurring in the polymerization process (Tan et al., 2021). Moreover, the FTIR spectrum of OPDOPA also exhibited remarkable differences compared with that of PDOPA. The stretching and bending vibrations of the N-H bond almost disappeared, whereas a new stretching vibration peak attributed to the C=N bond emerged at 1,552 cm^-1^. This observation demonstrates the transformation of C-N bonds into C=N bonds during oxidation. Additionally, the stretching vibration peak of the C=O bond (1,659 cm^-1^) appeared and was significantly intensified, suggesting that partial C-O bonds (1,093 cm^-1^) might be oxidized to C=O bonds. Meanwhile, the C-H stretching vibration peak of the benzene ring (3,220 cm^-1^) disappeared after oxidation, and the C=C skeletal vibration peaks of the aromatic ring (1,600–1,450 cm^-1^) were also absent. According to existing literature, OPDOPA exhibits a significant fluorescence activation during the oxidation process, which may be related to the formation of its conjugated skeletal structure. This structural evolution not only enhances planar rigidity but also effectively restricts low-frequency vibrational modes, thereby suppressing non-radiative decay and increasing the radiative decay rate (Hu et al., 2024).

XPS analysis was performed to characterize the elemental compositions and chemical states of OPDOPA and PDOPA. The full-range XPS spectra of OPDOPA and PDOPA (Figures 3G,H) exhibited three dominant peaks at 284.0, 400.0, and 531.0 eV, corresponding to C 1s, N 1s, and O 1s. The elemental contents of PDOPA were determined to be 70.06%, 8.72%, and 21.22%, while those of OPDOPA were 65.33%, 10.36%, and 24.30%. These results indicated that the elemental composition did not change significantly during the synthesis of OPDOPA. The relatively higher oxygen content in OPDOPA might be attributed to the residual H_2_O_2_ after the synthetic reaction.

**FIGURE 2:**
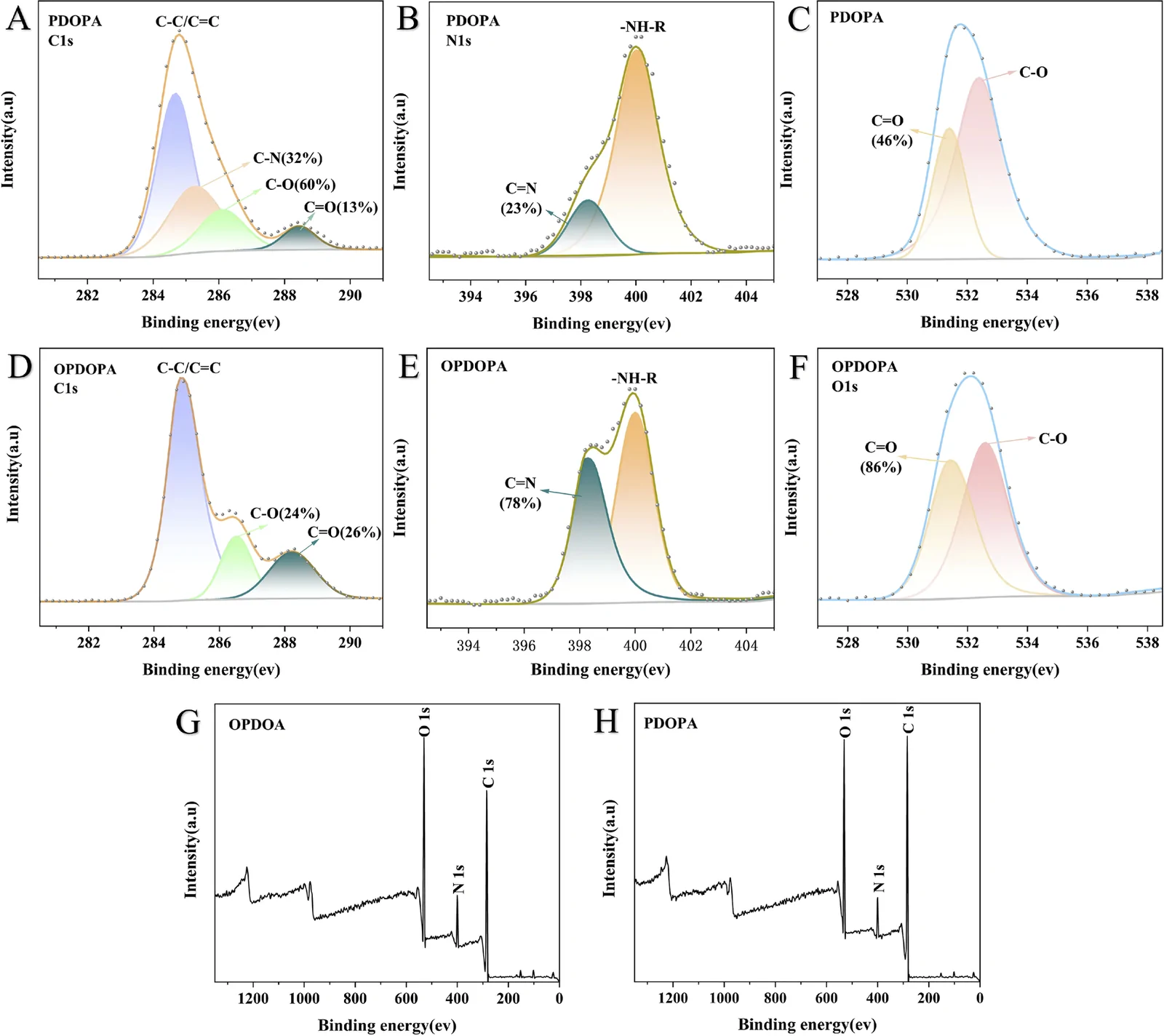
XPS spectra of C1s **(A)**, N1s **(B)**, and O1s **(C)** of PDOPA. XPS spectra of C1s **(D)**, N1s **(E)**, and O1s **(F)** of OPDOPA. The XPS spectrum of PDOPA **(G)** and OPDOPA **(H)**.

C 1s XPS spectra (Figures 2A,D) revealed four fitted peaks at 284.7, 285.3, 286.1, and 288.4 eV, assigned to the C−C/C=C, C−N, C−O, and C=O, respectively. The N 1s spectrum (Figures 2B,E) showed two peaks at 398.3 eV and 400.0 eV, corresponding to the C=N and N−H (Palsaniya and Mukherji, 2022). Two peaks centered at 531.4 eV and 532.4 eV were observed in O 1s (Figures 2C,F), which were associated with the C=O and C−O (Cheng et al., 2021). The variation tendency of functional groups revealed by XPS was consistent with that obtained from FT-IR spectroscopy. Specifically, the oxidation of PDOPA to OPDOPA resulted in elevated relative proportions of C=O and C=N, accompanied by a decrease in the contents of C−O and C−N. These findings provided unambiguous evidence for the oxidation reaction occurring during the formation of OPDOPA.

**FIGURE 3:**
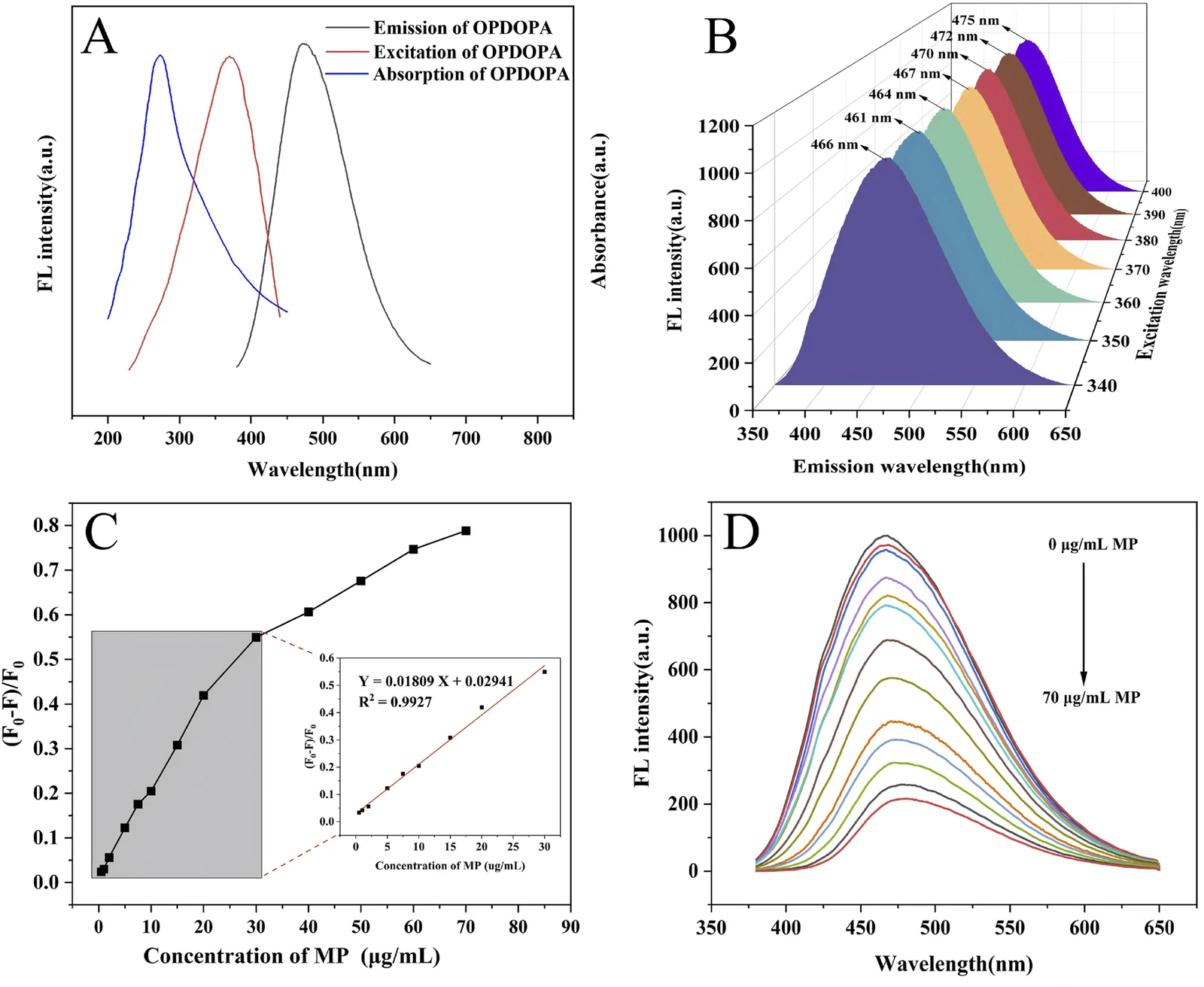
**(A)** UV-Vis absorption spectrum (blue), fluorescence excitation spectrum (red), and emission spectrum (black) of OPDOPA. **(B)** Fluorescence emission spectra of OPDOPA at varying excitation wavelengths. **(C)** Fitted scatter plots and (F_0_−F)/F_0_ of OPDOPA versus MP concentration. **(D)** Fluorescence spectra of OPDOPA after adding different concentrations of MP.

We characterized the optical properties of OPDOPA. As illustrated in Figure 3A, OPDOPA showed a UV-vis absorption band in the 200–300 nm region. Figure 3B presents the fluorescence emission spectra of OPDOPA recorded at excitation wavelengths ranging from 340 to 400 nm. The maximum fluorescence emission of OPDOPA was observed at 470 nm when excited at 370 nm. Notably, no significant red shift was detected with increasing excitation wavelength, suggesting that OPDOPA possesses a homogeneous particle size distribution. This finding is consistent with the particle size statistics derived from SEM characterization (Supplementary Figure S1).

### 3.2 Optimization of test conditions

Within the present work, the conversion efficiency of MP to p-NP plays a pivotal role in ensuring the accuracy of the proposed MP detection method. Thus, the key reaction parameters governing the hydrolysis process—pH, temperature, and reaction time—were comprehensively optimized.

The fluorescence quenching ratio (F_0_-F/F_0_) increased gradually with rising temperature, indicating enhanced hydrolysis efficiency of MP, and reached a plateau when the temperature exceeded 60 °C. Therefore, 60 °C was chosen as the optimal reaction temperature (Supplementary Figure S2A). As illustrated in Supplementary Figure S2B, the effect of hydrolysis time on the fluorescence quenching efficiency was investigated in detail at 60 °C. Similarly, (F_0_-F)/F_0_ increased gradually with prolonged reaction time and tended to level off at 40 min. For a more comprehensive assessment of how sodium hydroxide concentration influences the hydrolysis reaction, the (F_0_-F)/F_0_ under varying concentrations of sodium hydroxide was examined in detail (Supplementary Figure S2C). The fluorescence response intensity exhibited a positive correlation with increasing NaOH concentration and plateaued at 100 mM. Considering environmental friendliness and safety, 100 mM NaOH was used as the condition for the hydrolysis of MP. Finally, hydrolysis of MP was performed at 60 °C in 100 mM NaOH for 40 min to achieve complete hydrolysis. Furthermore, the incubation time between p-NP and OPDOPA after introducing OPDOPA into the detection system was optimized (Supplementary Figure S2D). The (F_0_-F)/F_0_ tended to stabilize after 5 min of co-incubation. Therefore, to minimize experimental errors, the subsequent detections were uniformly incubated for 10 min.

### 3.3 Linear range and selectivity

Under the optimal conditions, the fluorescence emission spectra of OPDOPA were recorded after co-incubation with various concentrations of MP, and the values of (F_0_-F)/F_0_ were calculated (Figure 3C). The fluorescence intensity of the OPDOPA solution decreased accordingly with the gradual increase of MP concentration (Figure 3D). The (F_0_-F)/F_0_ value exhibited a favorable linear correlation with MP concentration over the range of 0.5–30 μg/mL, as described by the equation: (F_0_−F)/F_0_ = 0.01809 C_MP_ (μg/mL) + 0.02941 (*R* ^*2*^ = 0.9927). Based on the formula LOD = 3S/K, the detection limit was calculated to be 0.08 μg/mL, with S denoting the standard deviation of the blank signal and K standing for the slope of the linear calibration curve.

To assess the selectivity of the fabricated sensor, various pesticides structurally similar to MP (phoxim, chlorpyrifos, dimethoate, fenthion, dichlorvos, methamidophos, and glyphosate) were examined. In Supplementary Figure S3, only MP (20 μg/mL) exhibited a significant fluorescence quenching effect on OPDOPA, whereas the interference caused by other pesticides (200 μg/mL) was negligible. Meanwhile, the hydrolysis product of MP, p-NP, exhibited a strong UV absorption at 403 nm, whereas none of the interfering pesticides showed obvious UV absorption at this wavelength when treated under the same conditions (Supplementary Figure S4). Therefore, the OPDOPA nanofluorescent sensor is immune to interference from other analogous pesticide residues, exhibiting high specificity for the detection of MP.

### 3.4 Stability of OPDOPA

The stability of OPDOPA was assessed via long-term tracking of the changes in fluorescence intensity. The fluorescence intensity of the OPDOPA solution stored at 4 °C was measured every 2 days. A 61.52% increase in fluorescence intensity was observed within 2 weeks, indicating that OPDOPA exhibits poor stability in aqueous solution (Supplementary Figure S5A). To address this issue, the OPDOPA solution was cryodesiccated to obtain a solid powder, which was then stored in a sealed vessel at −4.°C. After redissolution for testing, the fluorescence intensity of powdered OPDOPA only increased by 7.38% within 1 month (Supplementary Figure S5B). These results may be attributed to the removal of water and residual H_2_O_2_ during the lyophilization process, which reduces the occurrence of oxidation reactions and enhances stability (Cheng et al., 2016; Nowak and Jakubczyk, 2020). In addition, the powdered OPDOPA exhibits excellent solubility upon reconstitution.

### 3.5 MP detection mechanism

Common fluorescence quenching mechanisms include photoinduced electron transfer (PET), fluorescence resonance energy transfer (FRET), inner filter effect (IFE), dynamic quenching effect (DQE), and static quenching effect (SQE) (Li X. et al., 2025). As illustrated in Figure 3D, no obvious blue-shift or red-shift was observed in the fluorescence emission spectra with increasing MP concentration (0–70 μg/mL). This observation effectively eliminates the possibility of PET being involved in the quenching process (Zhang et al., 2023).

**FIGURE 4:**
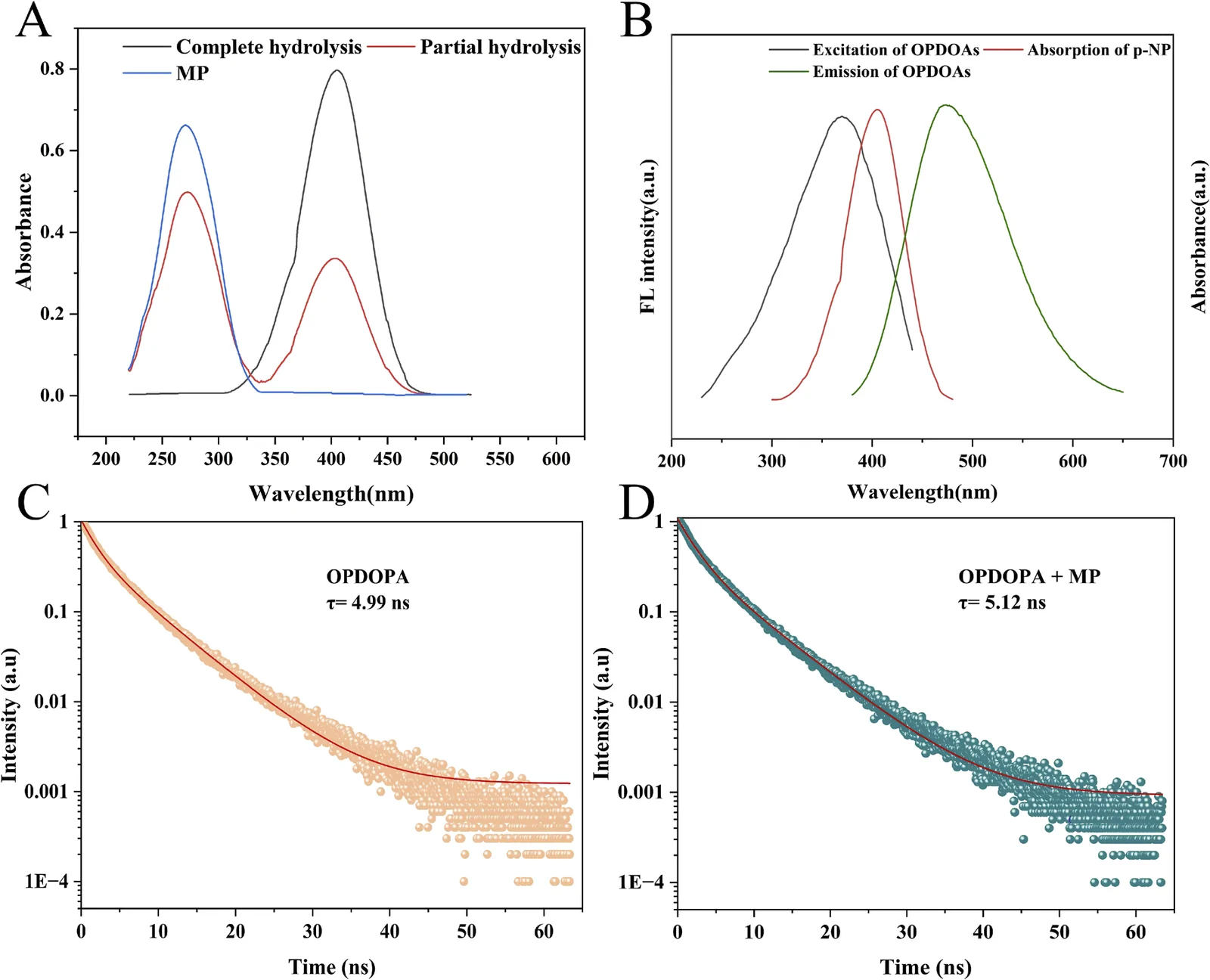
**(A)** UV absorption spectra of MP at different degrees of hydrolysis. **(B)** UV absorption spectrum of p-NP (red), the excitation (black) and emission (green) spectra of OPDOPA. **(C)** Fluorescence lifetime of OPDOPA. **(D)** Fluorescence lifetime of OPDOPA upon addition of MP.

Under highly alkaline conditions, MP undergoes hydrolysis to produce p-NP and O, O-dimethyl phosphorothioate (DMTP) (Yang et al., 2025). As evidenced by UV-Vis spectroscopy (Figure 4A), the UV spectrum of MP displays a prominent absorption band at 275 nm, while p-NP exhibits a strong absorption peak at around 400 nm; in contrast, DMTP shows no characteristic absorption peaks (Archana et al., 2020). The kinetic progress of MP hydrolysis was monitored via UV-Vis absorption spectroscopy. The results revealed that the absorption intensity of MP at 275 nm diminished progressively as the hydrolysis reaction proceeded, concomitant with a significant enhancement of the p-NP absorption peak at 403 nm. Under optimized hydrolysis conditions, the characteristic peak at 275 nm vanished completely, indicating the thorough conversion of MP to p-NP (Figure 4A). Further inspection demonstrated that the absorption spectrum of p-NP (centered near 400 nm) exhibits a substantial spectral overlap with the excitation (λ_ex_ = 370 nm) spectra of OPDOPA (Figure 4B). This spectral overlap characteristic strongly suggests that the fluorescence quenching mechanism is likely attributed to IFE (Zhang et al., 2023).

To gain insight into the fluorescence quenching mechanism of OPDOPA by p-NP, the fluorescence lifetimes of OPDOPA were compared in the absence and presence of p-NP (Figures 4C,D). The results show that the shape of the fluorescence decay curve was unaffected by the introduction of p-NP. Using the amplitude-weighted average lifetime equation (Equation 1), we calculated average lifetimes of 4.99 ns for pristine OPDOPA and 5.12 ns for the p-NP-treated sample. Notably, the average lifetime of OPDOPA increased by 1.25% after the introduction of p-NP, and the variation amplitudes of the two lifetime components (τ_1_ and τ_2_) were both less than 15%. No significant decrease in fluorescence lifetime was observed. These results indicate that there is no energy transfer between p-NP and OPDOPA, further confirming that the interaction between p-NP and OPDOPA is dominated by the IFE, which is consistent with previous studies based on the IFE mechanism (Zhang et al., 2019).

$$\tau =\frac{A_{1}\tau_{1\;}^{2\;}+A_{2}\tau_{2\;}^{2\;}}{A_{1}\tau_{1\;}+A_{2}\tau_{2\;}}$$ (1)

$$F_{\text{corr}}=F_{\text{obs}}\times 10^{\frac{A_{\text{ex}}+A_{\text{em}}}{2}}$$ (2)

Simultaneously, UV-Vis absorption spectroscopy was employed to verify whether the hydrolysis product of MP could form a ground-state complex with OPDOPA. As shown in Supplementary Figure S6, the UV-Vis absorption spectrum of the system exhibited no significant changes upon the addition of the hydrolysis product, indicating that it did not form a ground-state complex with OPDOPA. This observation effectively rules out the possibility of a static quenching mechanism. To verify the dominant role of IFE, the inner-filter correction was strictly performed according to the standard method (Cao et al., 2026; Kujur et al., 2024). As shown in Table 1, after correcting for the absorption of p-NP at both excitation and emission wavelengths using Equation 2, the corrected fluorescence intensity (F_corr_) remained essentially constant across the entire concentration range. The IFE contribution rates calculated at different concentrations were all greater than 90%. These quantitative results unequivocally demonstrate that the fluorescence decrease is predominantly governed by the inner filter effect, rather than by other mechanisms.

**TABLE 1:** Inner filter effect of p-NP on the fluorescence of OPDOPA.

p-NP concentration (μg/mL)	A_ex_	A_em_	F_obs_	F_corr_	IFE contribution rate (%)
0	0	0.000	743690	743690	-
2.5	0.048	0.005	697080	740939	94.10
5	0.115	0.009	638970	737022	93.63
10	0.234	0.014	548100	729222	92.60
20	0.477	0.026	403910	720750	93.25
40	0.974	0.047	228090	738935	99.08

### 3.6 Comparison of different methods

A comparative evaluation of the proposed sensor and other previously reported fluorescence-based detection strategies for MP is presented in Table 2. This comparison encompasses several critical analytical parameters, including the underlying detection mechanism, the category of fluorescent probe employed, the linear range, the LOD, and the type of real sample matrix investigated. The comparative data reveal that our method achieves a sensitivity that is largely commensurate with that of the majority of existing fluorescent sensors, while outperforming those operated via enzyme-inhibited or PET-mediated signal transduction pathways. In addition, the analytical performance of the present method is sufficient to fulfill the demands of practical residue monitoring. A noteworthy feature of this approach is that the entire detection process is accomplished through catalytic alkaline hydrolysis, obviating the need for any enzymatic reagents. This attribute endows the method with substantial practical advantages, including enhanced environmental sustainability, reduced assay cost, simplified operational procedures, and improved long-term stability. Collectively, these findings establish the proposed strategy as a promising new tool for the expeditious determination of MP residues.

**TABLE 2:** Comparison of this study with other MP detection methods.

Method	Probe	Linear range (μg/mL)	LOD (μg/mL)	Sample	References
MP hydrolase-catalyzed hydrolysis and IFE	N-doped carbon quantum dots (N-CQDs)	0.63–19.42	0.09	Water, soil, and pear	Song et al. (2017)
Alkaline hydrolysis and IFE	β-cyclodextrin-modified molybdenum disulfide quantum dots (β-CD-MoS_2_ QDs)	0.05–25	0.03	Apple and lettuce	Zhang et al. (2024)
Alkaline hydrolysis and IFE	silica oxide nanoparticles (SiONPs) conjugated with Eu^3+^	0.263–21.06	0.04	Rice	Chen et al. (2024)
Alkaline hydrolysis and IFE	Cu Nanoclusters (CuNCs)	0–6	0.04	Pear, cucumber, and soil	Li et al. (2022)
IFE	Covalent organic frameworks (COFs)	0.123–1.20	0.007	Water	Xiao et al. (2025)
Alkaline hydrolysis and PET	β-CD-MoS_2_ QDs	0.01–18.0	0.003	Apple and cucumber	Yi et al. (2021)
Alkaline hydrolysis and IFE	OPDOPA	0.5–30	0.08	*Codonopsis pilosula* and Potato	This work

### 3.7 Application in real sample analysis

To confirm the applicability of the proposed sensor in real sample analysis, spiked recovery tests with various concentration gradients were carried out in this study to assess its detection performance for *Codonopsis pilosula* and potato matrices. The spiked recoveries ranged from 92.00% to 106.42%, with the RSD less than 5.83% (Table 3). These results indicate that the as-constructed fluorescence sensor system exhibits favorable accuracy and stability in the analysis of two types of real samples.

**TABLE 3:** Detection of MP in *Codonopsis pilosula* and potato samples.

Sample	Spiked (mg/kg)	(F_0_-F)/F_0_	Detected (mg/kg)	Recovery Rate (%)	RSD (%, n = 3)
*Codonopsis pilosula*	0.01	0.0320	0.0094	93.70	4.47%
	0.02	0.0348	0.0199	99.68	4.86%
	0.04	0.0405	0.0408	101.96	4.52%
Potato	0.01	0.0319	0.0092	92.00	5.83%
	0.02	0.0352	0.0213	106.42	5.38%
	0.04	0.0405	0.0403	100.79	3.36%

## 4 Conclusion

In summary, this study successfully constructed a non-enzymatic fluorescent sensor with high sensitivity, environmental friendliness, low cost, excellent stability, and outstanding selectivity, which can be used for the rapid and efficient detection of MP in *Codonopsis pilosula* and potato. The OPDOPA prepared using L-DOPA as the initial material exhibits uniform particle size distribution and good dispersibility. Both the raw materials and the synthesis method adopted are environmentally friendly, conforming to the concept of ecological protection. The detection principle is based on the alkaline hydrolysis of MP to produce p-NP, and the fluorescence of OPDOPA is quenched via the IFE between p-NP and OPDOPA. This method exhibits high sensitivity and excellent stability in practical applications. The spiked recoveries in real samples ranged from 92.00% to 106.42%, with an RSD lower than 5.83%. It is suitable for the practical determination of MP in foods and traditional Chinese medicinal materials.

## Data Availability

The raw data supporting the conclusions of this article will be made available by the authors, without undue reservation.
