# Supplementary material for: Detection of methyl parathion using a novel oxidized polylevodopa fluorescence nanosensor
Source: Front Chem. 2026 Aug 6;14:1849994. doi: 10.3389/fchem.2026.1849994 (PMC13490100; doi:10.3389/fchem.2026.1849994)
Supplement: Supplementary file 1 [file DataSheet1.docx]

Supplementary Material

**Supplementary Figure 1.** (A) PDOPA particle size distribution histogram. (B) OPDOPA particle size distribution histogram.

**Supplementary Figure 2.** Effects of hydrolysis temperature (A), hydrolysis time (B), NaOH concentration (C) and incubation time (D) on the fluorescence quenching efficiency of OPDOPA

**Supplementary Figure 3.** Changes in the fluorescence intensity (F_0_/F) of OPDOPA induced by 20 μg/mL MP and 200 μg/mL of various interfering substances.

**Supplementary Figure 4.** UV absorption spectra of various organophosphorus pesticides after alkaline hydrolysis

**Supplementary Figure 5.**Variations in fluorescence emission intensity of OPDOPA with different storage times in liquid (A) and solid states (B)

**Supplementary Figure 6.** UV-Vis Absorption spectrum of OPDOPA, hydrolysis products ofMP, and their mixtures
